# Microfluidic Time-Delay Valve Mechanism on Paper-Based Devices for Automated Competitive ELISA

**DOI:** 10.3390/mi10120837

**Published:** 2019-11-30

**Authors:** Yu-Ting Lai, Chia-Hsin Tsai, Ju-Chun Hsu, Yen-Wen Lu

**Affiliations:** 1 Department of Biomechatronics Engineering, National Taiwan University, Taipei 10016, Taiwan; lukelai771230@hotmail.com; 2 Department of Entomology, National Taiwan University, Taipei 10016, Taiwan; r03632002@ntu.edu.tw (C.-H.T.); juchun@ntu.edu.tw (J.-C.H.)

**Keywords:** microfluidic valve, competitive enzyme-linked immunosorbent assay (ELISA), pesticide residue

## Abstract

Paper-based technologies have been drawing increasing attentions in the biosensor field due to their economical, ecofriendly, and easy-to-fabricate features. In this paper, we present a time-delay valve mechanism to automate a series of procedures for conducting competitive enzyme-linked immunosorbent assay (ELISA) on a paper-based device. The mechanism employs a controllable time-delay valve, which has surfactants to dissolve the hydrophobic barriers, in a fluid pathway. The valves can regulate the liquid and sequentially deliver the sample flow for automating ELISA procedures in microchannels. Competitive ELISA is achieved in a single step once the sample, or small molecule pesticide (e.g., Imidacloprid), is applied onto the paper-based device with a comparable sensitivity to plate-based competitive ELISA. The results further demonstrate the appositeness of using paper-based devices with the valve designs for on-the-go ELISA detection in agriculture and biomedical applications.

## 1. Introduction

As more evidence shows the relevance between pesticide exposure and birth defects, fetal death, and neurological disorder, the capability to detect the pesticide residue on foods or agricultural goods has become increasingly important [1,2,3]. Different detection methods for pesticide residence have thus been developed [4,5,6]. They however either are expensive or have small throughput, limiting their broader applications. Meanwhile, in biochemical analysis, paper-based devices have recently been popular [7,8,9,10,11]. Paper-based device for pesticide residue detection can be of great interest for on-the-go applications in agriculture and biomedical fields.

Pesticide residue detection today mainly relies on enzyme-linked immunosorbent assay (ELISA), the adaption to paper-based device is not straightforward nor trivial. ELISA is a sensitive method to measure the concentrations of antigen or antibody with high specificity. It typically involves a series of manual procedures—including mixing, washing, and incubation—which are sequentially conducted one by one. Few researchers have reported the ELISA application onto paper-based devices for biological testing as the procedures are tedious and time consuming, and become a major obstacle to be implemented onto a paper-based device [12,13]. A mechanism that permits these procedures to be performed on a paper-based device through time-delay valve designs will be necessary.

Various time-delay valve mechanisms have been developed to achieve sequential fluid flow deliveries by assigning different time delays to plural channels, which are formed by hydrophobic materials (e.g., wax) in geometrical patterns [14,15,16,17], on hydrophilic papers. Common examples are actuators [18,19,20,21], physical barriers [22,23,24,25,26,27], and chemical substances [28,29]. While they are all effective approaches, actuators require external force, and physical barriers may cause damage to paper-based devices. Therefore, dissolvable surfactant is utilized as the key component for our time-delay valve mechanism by reducing the surface tension of hydrophobic barriers.

To demonstrate the feasibility and broader applications of our mechanism on paper-based devices, the competitive ELISA is adapted, as it is perhaps one of the most complicated ELISA techniques. The competitive ELISA is a highly specific technique which is mostly utilized to measure the antigen concentration of a sample by detecting interference between target antibodies and antigens in an expected signal output. As the sample antigen concentrations increase, the output signals decrease, showing that the signal output inversely correlates with the amount of antigen in the sample. This is advantageous, particularly when detecting low concentration of samples or pesticide residue.

A single step automated competitive ELISA is achieved with our paper-based device. With the application of our unique time-delay valve mechanism to paper-based microchannels, undesirable multi-step reagent manipulations of plate-based competitive ELISA are eliminated. Imidacloprid, one of the most popular pesticides due to its high toxicity to insects but human [30,31], is tested with the device. The results reveal a comparable sensitivity to the data by conventional plate-based competitive ELISA.

## 2. Mechanism and Methods

**Figure 1:**
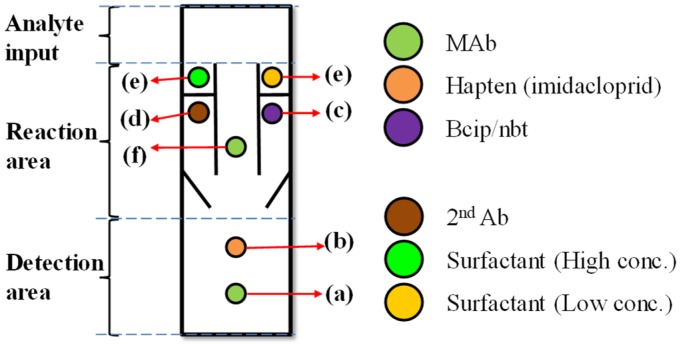
Schematic to show the paper-based enzyme-linked immunosorbent assay (ELISA) device with our time-delay valve mechanism. The device has three areas: analyte input, reaction area and detection area. The device is prepared before the detection with the following procedures: (**a**) MAb (antibody) is immobilized in the control area, (**b**) antigens specific to the target antibodies (Abs) is immobilized in the test area for colorimetric detection, (**c**) bcip/nbt solution to produce color, (**d**) enzyme-linked antibody (second Ab), (**e**) our time-delay valve design, which controls the timing and sequences of the liquid flow. (**f**) MAb combines with both the tested sample (imidacloprid) and antigen-competitive reaction.

A paper-based microfluidic device was constructed with a time-delay valve design to handle multiple fluids and to sequentially proceed the experimental steps of a competitive ELISA protocol, as shown in Figure 1. The device has three areas: analyte input, reaction, and detection areas. The analyte input area was where the analyte was added; it was designed with a certain size that could hold enough volume (e.g., 100 mL) for the sample solution to gradually diffuse into the second area—reaction area. The reaction area had three channels, including left, center, and right channels. The center channel had no hydrophobic wax barrier across the channel, so there was no time-delay valve to delay the flow that the solution could directly flow through. The antigen in the analyte (e.g., imidacloprid) meanwhile combined with the MAb (antibody) at (f) and the uncombined MAb were brought to the detection area along with the solution. The uncombined MAb combined with the antigen at (b). These were the competitive procedures between the antigen in the analyte and the antigen at (b). The right and left channels meanwhile were the delay channels. They had wax barriers, which functioned as time-delay valves, set across the channels to prevent the fluid wicking and to delay the solution passing through. The detection area included two areas: (a) control area, which contained immobilized antibodies (Abs) for enzyme-linked detection, and (b) test area, which contained the immobilized specific antigens to the target Abs.

**Figure 2:**
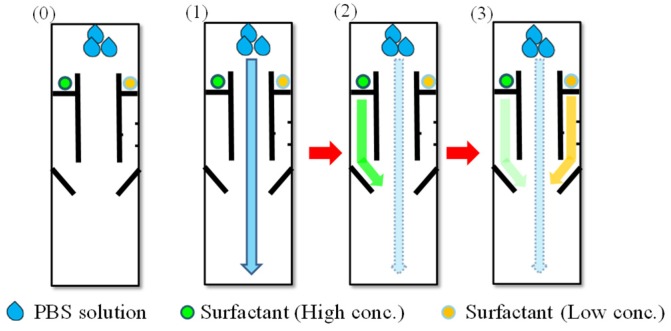
Schematic to show the time-delay valve mechanism in three steps: (**0**) The device is made before the solutions flow through the channels. (**1**) The solution first flows through the central channel. (**2**,**3**) The left and the right channels are sequentially opened by the solution, which dissolves surfactant at different concentrations.

The time-delay valve was employed to sequentially control the flow passing through these three channels. As shown in Figure 2, once the device was made, the analyte was first added in the input area and flowed into the three channels in the reaction area. While the analyte solution could pass through the center channel quickly, it took much longer time to pass through the side channels. The solution dissolved the surfactant and became capable of reducing the surface tension of the wax barrier, which promoted its wicking ability, so the solution could penetrate the barrier and pass through the side channels. This is the time-delay valve mechanism. The time duration for solution to pass each time-delay valve was determined by the wax-barrier width and the surfactant concentration. Such a mechanism can permit the sequential delivery of the enzyme-linked second Ab in the left channel and bcip/nbt solution in the right channel to the detection area. Once the solutions reached the detection area and mixed antigen (in test area) and MAb (in control area) for the enzyme reactions taking places. A colored product was then formed. A visible color change can be observed in the detection area.

As for the design pattern, a multiple step process was performed sequentially by three steps with time-delay valve mechanism: (1) The central sample fluid laterally flowed through the reaction area to detection area without barrier, resulting in the competitive reaction with the pre-spotted MAb between the analyte and the immobilized antigens. The side fluids dissolved the pre-spotted surfactant solutions at different concentrations. (2) It took less time for the left side fluid flow (higher surfactant concentration) to penetrate the wax barrier and transport the second Ab to the test area for capture of enzyme-second Ab. (3) On the other hand, it took longer time for the right side fluid flow (lower surfactant concentration) to penetrate and transport the bcip/nbt substrate to the detection area. The enzyme reaction then occurred at the test area to produce a visible color change. These were the procedures of competitive ELISA.

## 3. Device Fabrication

### 3.1. Device Fabrication

The patterns in Figure 1 were designed with AutoCAD (Autodesk, Inc., San Rafael, CA, USA) and printed on an NC membrane (nitrocellulose membrane) with a solid ink printer (Xerox, ColorQube8580, Xerox, Norwalk, CA, USA) using solid wax ink (Xerox, Genuine Solid Ink Black). After the wax was printed, the membrane was baked at 125 °C for 120 s to melt the wax into the membrane to form hydrophobic patterns.

### 3.2. Preparation of the Paper-Based Enzyme-Linked Immunosorbent Assay (ELISA) Device for the Detection of Imidacloprid

In our ELISA protocol, the monoclonal mouse anti-imidacloprid antibody (MAb) and imidacloprid-antigen (antigen) solutions (0.5 µL at 1 mg/mL) were spotted on the NC membrane in the control and test areas at locations (a,b) in Figure 1. After drying for 1 h at room temperature, the membrane was blocked to against nonspecific protein adsorption in bovine serum albumin (BSA) solution for 1 h. The bcip/nbt substrate, polyclonal goat anti-mouse IgG conjugated with alkaline phosphatase (second Ab) and the MAb were spotted by a micropipette onto the membrane at locations (c,d,f), respectively. After drying the membrane for 1 h at room temperature, different concentrations of surfactant solution (e.g., Tween 20) was dispensed on the membrane at location (e).

### 3.3. Materials

Monoclonal mouse anti-imidacloprid antibody (imidacloprid-MAb) and imidacloprid-antigen were obtained from Department of Entomology, National Taiwan University (Taipei, Taiwan). Polyclonal goat anti-mouse IgG conjugated with Alkaline phosphatase (AP) were purchased from Invitrogen (Invitrogen, Carlsba, CA, USA). The bcip/nbt (5-bromo-4-chloro-39-indolyphosphate p-toluidine salt, nitro-blue tetrazolium chloride) substrate solution and substrate buffer solution was purchased from Nacalai Tesque (Taipei, Taiwan). Bovine serum albumin (BSA), phosphate-buffered saline (PBS), polysorbate 20 (Tween 20), and p-nitrophenyl phosphate (pNPP) were purchased from Sigma-Aldrich (St. Louis, MI, USA).

### 3.4. Data Processing

A smartphone camera, Sony z1 (20.7 megapixel, Sony Corporation, Tokyo, Japan), was used to capture images of the competitive ELISA results. The color intensity of the image was quantified by an image processing program (ImageJ, National Institutes of Health, Bethesda, MD, USA). Quantified data from the test and control areas were subtracted by the background data, acquired from the area above the test area, for calibration.

## 4. Result and Discussion

### 4.1. Time-Delay Valve

**Figure 3:**
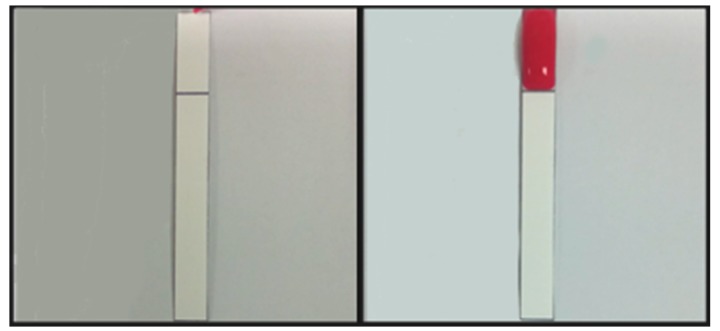
Barrier penetration test. (**Left**): Surfactant solution in 0.5 μL was added at the front of the barrier and dried for 1 h; (**Right**): phosphate-buffered saline (PBS) (100 μL) was added for penetration.

Surfactant, surfactant concentration, and wax barrier width were the main factors that affected the performance of the time-delay valve. Tween 20 and Triton X-100, gentle surfactants which were common agents for immunoblotting and ELISA, were used to enable the solution to penetrate the hydrophobic wax barrier by lowering the surface tension of the barrier. Several combinations of different wax barrier widths, and surfactant concentrations were tested for finding the optimal parameters for our device. During the tests, surfactants at different concentrations in 0.5 μL were dispensed at the front of wax barrier and dried for 1 h for complete evaporation (Figure 3 left). PBS was added (100 μL) to dissolve the surfactant for penetrating the wax barrier.

**Figure 4:**
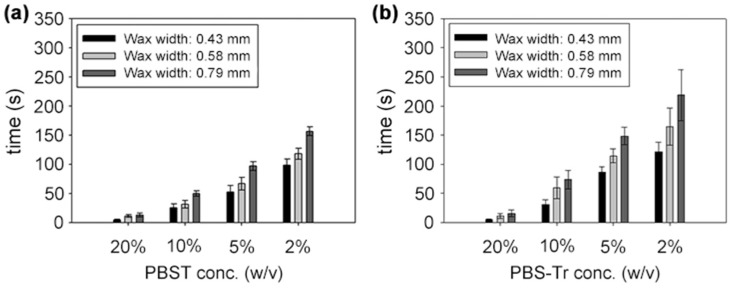
Time required for the solutions to penetrate the wax barriers when different concentrations of (**a**) PBST and (**b**) PBS-Tr and different barrier widths were applied. Experiments were conducted at least three times for each concentration of both surfactants. (**a**) is reproduced with permission from reference [32].

Figure 4 showed the effects of surfactant, surfactant concentration, and barrier width on the time for the solutions to fully penetrate the barrier. It took less time as the surfactant concentration increased, while it took more time as the barrier width increased. It took much longer time for the PBS-Tr (Triton X-100 in PBS solution) to pass through the wax barrier than PBST (Tween 20 in PBS solution) did.

**Figure 5:**
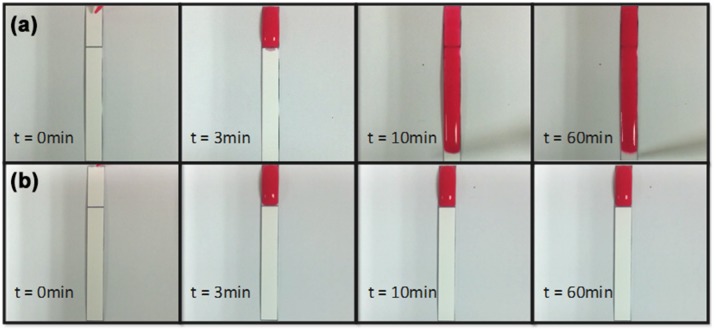
Sequence photography of PBS-surfactant penetration tests. (**a**) 2% surfactant solution pre-spotted; (**b**) surfactant solution at concentration lower than 2% (e.g., 1%) pre-spotted.

In Figure 5a, PBS-surfactant (2%) started penetrating through the barrier 3 min after the dispensation of PBS. Figure 5b showed that PBS-surfactant at concentration lower than 2% could not penetrate through the barriers one hour after the dispensation of PBS.

Tests were conducted to find the optimal delaying time for each time-delay valve to sequentially control the flow passing through all three channels on our device. It was found that delaying time less than 30 s resulted in few differences between the arrival of flows from side and central channels in the detection area, and delaying time longer than 180 s was too long that the sample evaporated. As a result, time-delay valves with delaying time ranged from 30–60 s was adapted to sequentially deliver second Ab and bcip/nbt substrate from the side channels on our device.

**Figure 6:**
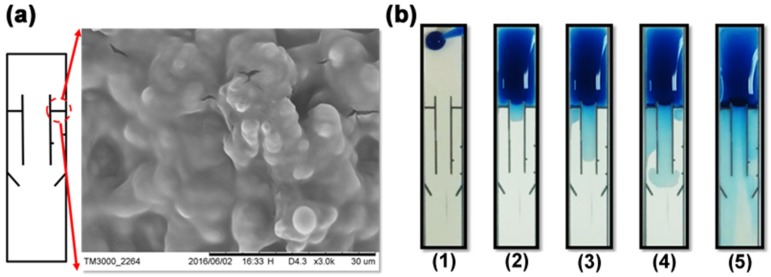
(**a**) The inset showed the SEM image of the wax barrier. The barrier can be penetrated by the sample-surfactant flow, allowing the flow to pass through the side channel. (**b**) The image sequences showed the five steps in the time-delay valve while each step was approximately 30–60 s before the next step. These five steps are: (**1**) The sample was added on the input area. (**2**) The sample laterally flowed through the reaction area to the detection area in the central channel, triggering the competitive reaction. (**3**) The left time-delay valve was opened and the second-Ab-carried solution flowed through the left side channel. (**4**) The right time-delay valve was opened and the bcip/nbt-substrate-carried solution flowed through the right-side channel. (**5**) All solutions (e.g., Mab, second Ab, and bcip/nbt) arrived in the detection area, which triggered the enzyme reaction. (**a**) is reproduced with permission from reference [32].

Figure 6a showed the SEM image of the wax barrier. The barrier prevents the sample flow from passing through the side channel unless there are surfactants, which inhibits the hydrophobicity of the barrier. In Figure 6b, surfactant solution was properly pre-spotted on both side channels (e.g., 10% on the left side channel and 5% on the right one). The sample flows dissolved the pre-spotted surfactant on both side channels and penetrated through the barriers with different timed durations (e.g., 30–60 s), resulting in the sequential arrival of the solutions (e.g., MAb, second Ab, and bcip/nbt) in the detection area.

### 4.2. Performance of Autonomous Paper-Based Devices

**Figure 7:**
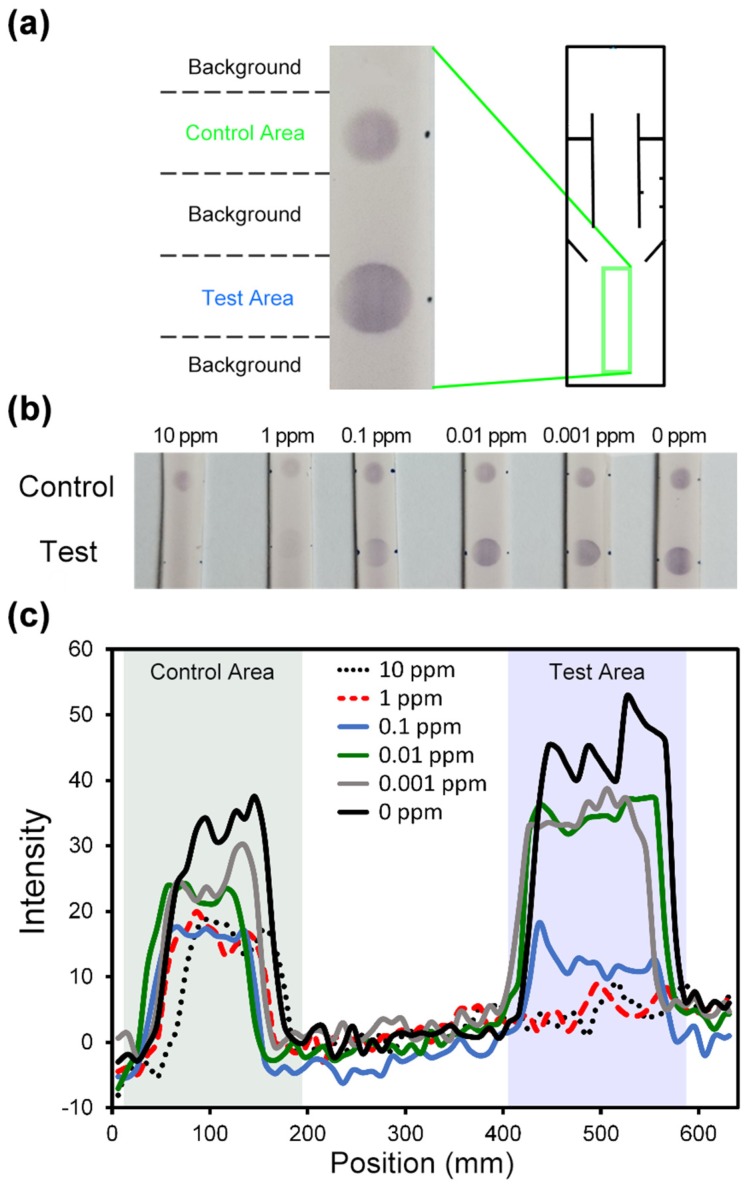
(**a**) The images of the chromogenic signals from the detection area in our paper-based device. Cycle T represented the test area while Cycle C represented the control area. (**b**) The images of our paper-based device in the detection area, after the competitive ELISA procedures were conducted for different sample concentrations. (**c**) The color intensities in the detection area are quantified, along with the locations.

Competitive ELISA for Imidacloprid detection was conducted. In Figure 7a, the chromogenic signals, which responded to the concentrations of imidacloprid, in the detection area were acquired with a smartphone camera (Sony z1).

Figure 7b illustrated the results of competitive ELISA testing samples at different concentrations on our devices. The tested sample and immobilized antigen competitively bound to the antibody (pre-spotted MAb): The more antigen in the sample, the less antibody to bind to the immobilized antigen—competitive reaction. Second Ab and bcip/nbt substrate solution arrived in the detection area sequentially from the side channels. The enzyme reaction happened at the detection area, which elicited a chromogenic signal—a visible color change, which increased as the sample concentration decreased.

The color changes triggered by the enzyme reaction were quantified using Image-J. The quantification data were shown in Figure 7c, which indicated that the color intensity increased in the test area as the sample concentrations decreased. The 0 ppm sample resulted in the largest color change, while the 1 ppb, 10 ppb, and 0.1 ppm samples made noticeable color changes. The 1 ppm and 10 ppm samples however had very few color changes. The results indicated that our device was applicable to imidacloprid detection with a detection limit at the sample concentration of 0.1 ppm.

The data were normalized in order to compare to the results by plate-based ELISA. Normalized color intensity (T/C) was defined as

(1) $$T/C=\frac{T_{i}-T_{min}}{C-T_{min}}$$

where $T_{i}$ was the color intensity in the test area, $T_{min}$ was the minimum color intensity in the test area. C was the color intensity in the control area.

**Figure 8:**
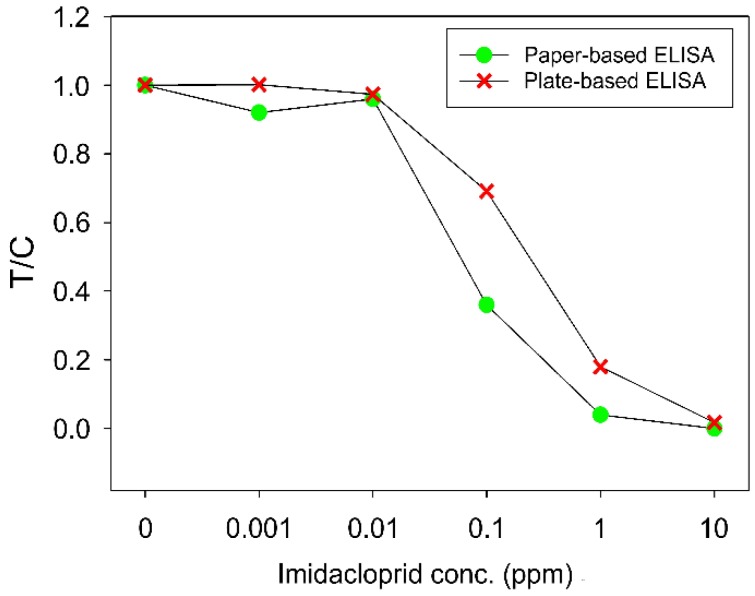
*T*/*C* values from paper-based and plate-based competitive ELISA that tested Imidacloprid samples at different concentrations. Each data point is the average value from three measurements.

Figure 8 showed that the T/C values decreased as the sample concentrations increased in both paper-based and plate-based ELISA. Paper-based and plate-based ELISA, which detected Imidacloprid at the concentrations of 0, 0.001, 0.01, 0.1, 1, and 10 ppm. Both types of ELISA resulted in similar results, which validated the applicability of our devices to on-site pesticide detection. The color intensities of the test areas, from the photos of the paper-based device, acquired with a mobile phone, can be analyzed for concentration quantification.

Finally, as conventional ELISAs are conducted in plates with multiple steps and expensive spectrophotometric readers to quantify the biomarkers, this automated paper-based ELISA analysis device is very useful as a single-step economic analysis tool and potentially compatible with digital or cellular phone cameras. It can provide considerable impact, in particular for daily life or agriculture applications.

## 5. Conclusions

A time-delay valve mechanism on a paper-based device is developed to automate the competitive ELISA procedures. The time-delay valve mechanism is applicable to sequentially delivering fluid flows for a series of reaction steps. Multiple-step competitive ELISA for Imidacloprid detection is successfully conducted with a similar sensitivity to the ELISA procedures done in plates. The device can be further improved and extended its capability of performing detection for clinical or environmental analytes [33,34,35,36].

## References

[1] Alavanja M.C., Hoppin J.A., Kamel F. (2004). Health effects of chronic pesticide exposure: Cancer and neurotoxicity. Annu. Rev. Public Health.

[2] Kamel F., Hoppin J.A. (2004). Association of pesticide exposure with neurologic dysfunction and disease. Environ. Health Perspect..

[3] Gilden R.C., Huffling K., Sattler B. (2010). Pesticides and health risks. J. Obstet. Gynecol. Neonatal Nurs..

[4] Navalon A., Gonzalez-Casado A., El-Khattabi R., Vilchez J.L., Fernández-Alba A.R. (1997). Determination of imidacloprid in vegetable samples by gas chromatography–mass spectrometry. Analyst.

[5] MacDonald L.M., Meyer T.R. (1998). Determination of imidacloprid and triadimefon in white pine by gas chromatography/mass spectrometry. J. Agric. Food Chem..

[6] Baskaran S., Kookana R.S., Naidu R. (1997). Determination of the insecticide imidacloprid in water and soil using high-performance liquid chromatography. J. Chromatogr. A.

[7] Lepowsky E., Ghaderinezhad F., Knowlton S., Tasoglu S. (2017). Paper-based assays for urine analysis. Biomicrofluidics.

[8] Li C.Z., Vandenberg K., Prabhulkar S., Zhu X., Schneper L., Methee K., Almeide E. (2011). Paper based point-of-care testing disc for multiplex whole cell bacteria analysis. Biosens. Bioelectron..

[9] Li X., Ballerini D.R., Shen W. (2012). A perspective on paper-based microfluidics: Current status and future trends. Biomicrofluidics.

[10] Xia Y., Si J., Li Z. (2016). Fabrication techniques for microfluidic paper-based analytical devices and their applications for biological testing: A review. Biosens. Bioelectron..

[11] Kung C.T., Hou C.Y., Wang Y.N., Fu L.-M. (2019). Microfluidic paper-based analytical devices for environmental analysis of soil, air, ecology and river water. Sens. Actuators B Chem..

[12] Cheng C.M., Martinez A.W., Gong J., Mace C.R., Phillips S.T., Carrilho E., Whitesides G.M. (2010). Paper-based ELISA. Angew. Chem..

[13] Wang S., Ge L., Song X., Yu J., Ge S., Huang J., Zeng F. (2012). Paper-based chemiluminescence ELISA: lab-on-paper based on chitosan modified paper device and wax-screen-printing. Biosens. Bioelectron..

[14] Apilux A., Ukita Y., Chikae M., Chailapakul O., Takamura Y. (2013). Development of automated paper-based devices for sequential multistep sandwich enzyme-linked immunosorbent assays using inkjet printing. Lab Chip.

[15] Carrilho E., Martinez A.W., Whitesides G.M. (2009). Understanding wax printing: a simple micropatterning process for paper-based microfluidics. Anal. Chem..

[16] Fu E., Lutz B., Kauffman P., Yager P. (2010). Controlled reagent transport in disposable 2D paper networks. Lab Chip.

[17] Juncker D., Schmid H., Drechsler U., Wolf H., Wolf M., Michel B., Delamarche E. (2002). Autonomous microfluidic capillary system. Anal. Chem..

[18] Li X., Tian J., Nguyen T., Shen W. (2008). Paper-based microfluidic devices by plasma treatment. Anal. Chem..

[19] Martinez A.W., Phillips S.T., Nie Z., Cheng C.M., Carrilho E., Wiley B.J., Whitesides G.M. (2010). Programmable diagnostic devices made from paper and tape. Lab Chip.

[20] Shin J.H., Park J., Kim S.H., Park J.K. (2014). Programmed sample delivery on a pressurized paper. Biomicrofluidics.

[21] Wang X., Hagen J.A., Papautsky I. (2013). Paper pump for passive and programmable transport. Biomicrofluidics.

[22] Chumo B., Muluneh M., Issadore D. (2013). Laser micromachined hybrid open/paper microfluidic chips. Biomicrofluidics.

[23] Gaspar C., Sikanen T., Franssila S., Jokinen V. (2016). Inkjet printed silver electrodes on macroporous paper for a paper-based isoelectric focusing device. Biomicrofluidics.

[24] Giokas D.L., Tsogas G.Z., Vlessidis A.G. (2014). Programming fluid transport in paper-based microfluidic devices using razor-crafted open channels. Anal. Chem..

[25] He P.J.W., Katis I.N., Eason R.W., Sones C.L. (2015). Engineering fluidic delays in paper-based devices using laser direct-writing. Lab Chip.

[26] Oyola-Reynoso S., Frankiewicz C., Chang B., Chen J., Bloch J.F., Thuo M.M. (2017). Paper-based microfluidic devices by asymmetric calendaring. Biomicrofluidics.

[27] Yakoh A., Chaiyo S., Siangproh W., Chailapakul O. (2019). 3D Capillary-driven paper-based sequential microfluidic device for electrochemical sensing applications. ACS Sens..

[28] Lutz B., Liang T., Fu E., Ramachandran S., Kauffman P., Yager P. (2013). Dissolvable fluidic time delays for programming multi-step assays in instrument-free paper diagnostics. Lab Chip.

[29] Chen H., Cogswell J., Anagnostopoulos C., Faghri M. (2012). A fluidic diode, valves, and a sequential-loading circuit fabricated on layered paper. Lab Chip.

[30] Eddleston M., Bateman D.N. (2012). Pesticides. Medicine.

[31] Elbert A., Becker B., Hartwig J., Erdelen C. (1991). Imidacloprid-a new systemic insecticide. Pflanzenschutz Nachr. Bayer (Ger. FR).

[32] Lai Y.T., Tsai J.S., Hsu J.C., Lu Y.W. Automated paper-based devices by microfluidic timing-valve for competitive ELISA. Proceedings of the IEEE 30th International Conference on Micro Electro Mechanical Systems (MEMS).

[33] Cai L., Xu C., Lin S., Luo J., Wu M., Yang F. (2014). A simple paper-based sensor fabricated by selective wet etching of silanized filter paper using a paper mask. Biomicrofluidics.

[34] Kumar S., Bhushan P., Krishna V., Bhattacharya S. (2018). Tapered lateral flow immunoassay based point-of-care diagnostic device for ultrasensitive colorimetric detection of dengue NS1. Biomicrofluidics.

[35] Li H., Han D., Pauletti G.M., Steckl A.J. (2018). Engineering a simple lateral flow device for animal blood coagulation monitoring. Biomicrofluidics.

[36] Syms R. (2017). Rapid evaporation-driven chemical pre-concentration and separation on paper. Biomicrofluidics.

